# Superoxide Radical Metabolism in Sweet Pepper (*Capsicum annuum* L.) Fruits Is Regulated by Ripening and by a NO-Enriched Environment

**DOI:** 10.3389/fpls.2020.00485

**Published:** 2020-05-14

**Authors:** Salvador González-Gordo, Marta Rodríguez-Ruiz, José M. Palma, Francisco J. Corpas

**Affiliations:** Group of Antioxidants, Free Radicals and Nitric Oxide in Biotechnology, Food and Agriculture, Department of Biochemistry, Cell and Molecular Biology of Plants, Estación Experimental del Zaidín, Spanish National Research Council (CSIC), Granada, Spain

**Keywords:** NADPH oxidase, nitric oxide, nitration, pepper fruit, respiratory burst oxidase homolog, *S*-nitrosation, superoxide dismutase, ripening

## Abstract

Superoxide radical (O_2_^•–^) is involved in numerous physiological and stress processes in higher plants. Fruit ripening encompasses degradative and biosynthetic pathways including reactive oxygen and nitrogen species. With the use of sweet pepper (*Capsicum annuum* L.) fruits at different ripening stages and under a nitric oxide (NO)-enriched environment, the metabolism of O_2_^•–^ was evaluated at biochemical and molecular levels considering the O_2_^•–^ generation by a NADPH oxidase system and its dismutation by superoxide dismutase (SOD). At the biochemical level, seven O_2_^•–^-generating NADPH-dependent oxidase isozymes [also called respiratory burst oxidase homologs (RBOHs) I–VII], with different electrophoretic mobility and abundance, were detected considering all ripening stages from green to red fruits and NO environment. Globally, this system was gradually increased from green to red stage with a maximum of approximately 2.4-fold increase in red fruit compared with green fruit. Significantly, breaking-point (BP) fruits with and without NO treatment both showed intermediate values between those observed in green and red peppers, although the value in NO-treated fruits was lower than in BP untreated fruits. The O_2_^•–^-generating NADPH oxidase isozymes I and VI were the most affected. On the other hand, four SOD isozymes were identified by non-denaturing electrophoresis: one Mn-SOD, one Fe-SOD, and two CuZn-SODs. However, none of these SOD isozymes showed any significant change during the ripening from green to red fruits or under NO treatment. In contrast, at the molecular level, both RNA-sequencing and real-time quantitative PCR analyses revealed different patterns with downregulation of four genes *RBOH A*, *C*, *D*, and *E* during pepper fruit ripening. On the contrary, it was found out the upregulation of a *Mn-SOD* gene in the ripening transition from immature green to red ripe stages, whereas a *Fe-SOD* gene was downregulated. In summary, the data reveal a contradictory behavior between activity and gene expression of the enzymes involved in the metabolism of O_2_^•–^ during the ripening of pepper fruit. However, it could be concluded that the prevalence and regulation of the O_2_^•–^ generation system (NADPH oxidase-like) seem to be essential for an appropriate control of the pepper fruit ripening, which, additionally, is modulated in the presence of a NO-enriched environment.

## Introduction

Fruit ripening is a genetically coordinated developmental process that involves important physiological and biochemical changes affecting their organoleptic properties such as color, flavor, aroma, texture, and nutritional quality (Giovannoni, 2001; Osorio et al., 2013; Karlova et al., 2014). The ripening process occurs naturally when the fruits are in the plant, but it can also take place during the period of postharvest and storage especially in those types of fruits whose ripening depends on ethylene (climacteric fruits), such as tomato (*Solanum lycopersicum* L.), custard apple (*Annona squamosa* L.), banana (*Musa* spp.), or mango (*Mangifera indica* L.).

Pepper (*Capsicum* spp.) fruit, a non-climacteric one, belongs to the Solanaceae family, which includes other members such as tomatoes, potatoes, or eggplant. Fresh or processed pepper fruits are widely consumed around the world, so they have a great economic importance. Pepper fruit including many types and varieties differ in size, shape, color, and degree of pungency (hotness), being this last feature due to the presence of capsaicin (8-methyl-*N*-vanillyl-trans-6-nonenamide), and an alkaloid produced as a secondary metabolite. Furthermore, pepper fruits contain a high level of vitamin C and other vitamins such as A, E, B1, and B2 (Rodríguez-Ruiz et al., 2017). Pepper fruit ripening has an associated drastic change in color because the initial green color due to chlorophylls is progressively decomposed and is substituted by a different range of carotenoids including β-carotene, lutein, violaxanthin, capsanthin, violaxanthin, and zeaxanthin among others, which contribute to provide a variety of color (red, yellow, orange, purple/violet, etc.) in the ripe stage (Pascual et al., 2010; Gómez-García and Ochoa-Alejo, 2013; Ou et al., 2013; Liu et al., 2020). In fact, this variety of color and shape is even being used for ornamental purposes. In general, the main function of these carotenoids is the protection against oxidative damage because they can interact with singlet oxygen (^1^O_2_) as well as scavenger of peroxy radicals (LOOŢ), but also they are precursors of phytohormones such as abscisic acid (ABA) or strigolactones (Fanciullino et al., 2014).

Reactive oxygen and nitrogen species (ROS/RNS) are two families of molecules that are part of the physiological metabolism in all types of cells. Some of these molecules such as hydrogen peroxide (H_2_O_2_) or nitric oxide (NO) have regulatory functions in many physiological processes including seed germination, plant growth and development, and senescence, but also these reactive species could be also overproduced under adverse environmental conditions being involved in nitro-oxidative processes (Corpas and Barroso, 2013). Recently, biochemical evidences support that both ROS and RNS interact and seem to be involved in fruit ripening (Tanou et al., 2015; Palma et al., 2015, 2019; Corpas et al., 2018; Corpas and Palma, 2018; Léchaudel et al., 2018; Rodríguez-Ruiz et al., 2019).

NADPH oxidase (NOX), also called respiratory burst oxidase homolog (RBOH), is one of the main enzymatic sources of superoxide radicals (O_2_^•–^) in plants (Sagi and Fluhr, 2006; Suzuki et al., 2011; Marino et al., 2012). RBOHs participate in a wide range of function including cellular signal transduction, cell growth and development, pollen germination and tube growth, stomatal movements, plant interaction with beneficial organism, plant defense against pathogens (Müller et al., 2009; Jiménez-Quesada et al., 2016, 2019; Qu et al., 2017; Wang et al., 2018; Fonseca-García et al., 2019; Kaya et al., 2019). The number of *RBOH* genes reported thus far differs among the different plant species. Thus, 10 *RBOH* genes have been identified in the model plant *Arabidopsis*, nine in rice (*Oryza sativa*) (Wang et al., 2013; Kaur and Pati, 2018) and common bean (*Phaseolus vulgaris*) (Montiel et al., 2012), eight in tomato (*Solanum lycopersicum*) (Li et al., 2015) and seven in strawberry (*Fragaria* × *ananassa* cv. Toyonaka) (Zhang et al., 2018), grape (*Vitis vinifera*) (Cheng et al., 2013), and pepper (*Capsicum annuum* L.). This diversity of *RBOH* genes suggests that the corresponding proteins could have specific function according to their organ location (root, stem, leaf, flower, or fruit), physiological stage (seedling or flowering plants), or environmental conditions.

Superoxide dismutases (SODs) are metalloenzymes that catalyze the disproportionation of O_2_^•–^ into O_2_ and H_2_O_2_ and are associated with stress, development, and senescence conditions in plants (Houmani et al., 2016; del Río et al., 2018). Moreover, the type and number of SOD isozymes can differ between different plant species and their corresponding organs (Asada et al., 1973; Bridges and Salin, 1981; Almansa et al., 1989; Corpas et al., 1991, 2006; Pinilla et al., 2019). In preliminary studies, the presence of four SOD and seven O_2_^•–^-generating NOX isozymes, which seemed to be differentially regulated during ripening in different sweet pepper cultivars, was reported (Mateos et al., 2013; Chu-Puga et al., 2019). Moreover, additional research has identified and characterized other enzymatic and non-enzymatic antioxidant systems in pepper fruit (Jiménez et al., 2003; Mateos et al., 2009; Martí et al., 2009, 2011; Camejo et al., 2015; Chiaiese et al., 2019; Rodríguez-Ruiz et al., 2019; Ribes-Moya et al., 2020). With the goal to get a deeper knowledge of the physiological function and the regulation of the O_2_^•–^ metabolism in the ripening of sweet pepper fruit, biochemical and molecular approach-based in-gel activity assays and RNA-Seq/real-time quantitative PCR (RT-qPCR) analyses were accomplished in fruits at different ripening stages and in the presence of a NO-enriched environment. The data provide differential profile between biochemical and molecular results; however, it can be concluded that during ripening of sweet pepper from green to red stage, there is a prevalence of O_2_^•–^ generation, which seems to be negatively modulated in the presence of a NO-enriched environment.

## Materials and Methods

### Plant Material and Nitric Oxide Gas Treatment

**FIGURE 1:**
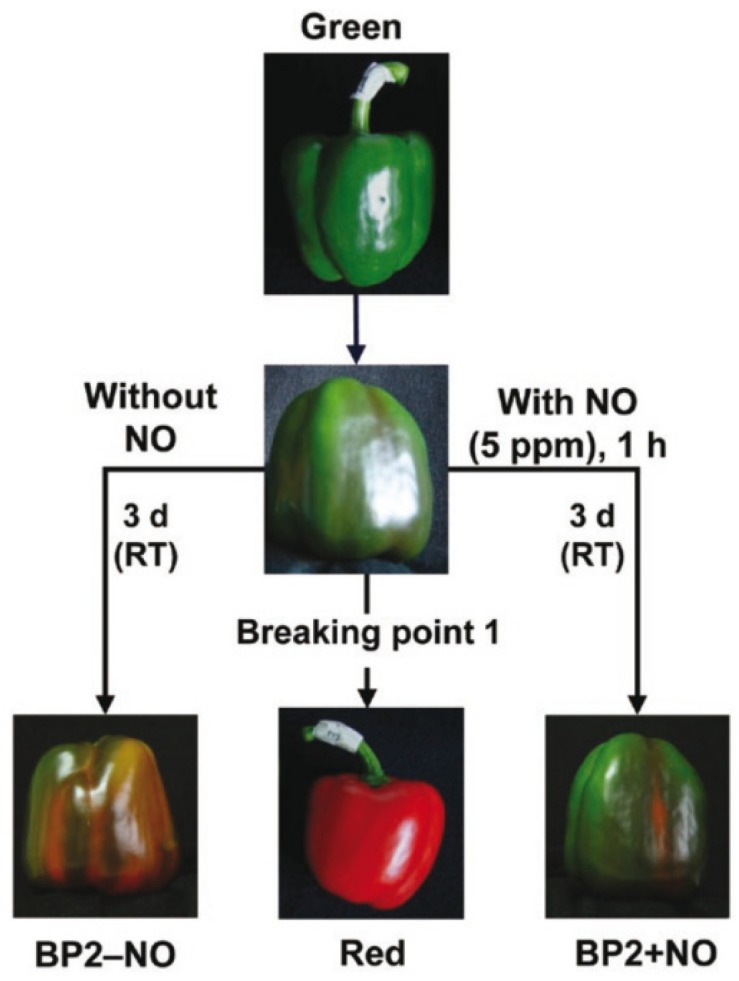
Illustrative picture showing the experimental design used in this study with the representative phenotype of sweet pepper (*Capsicum annuum* L.) fruits at different stages and treatments: immature green, breaking point 1 (BP1), breaking point 2 without NO treatment (BP2 – NO), breaking point 2 with NO treatment (BP2 + NO), and ripe red. Fruits were subjected to a NO-enriched atmosphere (5 ppm) in a hermetic box for 1 h and were then stored at room temperature (RT) for 3 days. Reproduced with permission from González-Gordo et al. (2019).

California-type sweet pepper (*Capsicum annuum* L., cv. Melchor) fruits were collected at three different developmental stages – green immature (G), breaking point (BP1), and red ripe (R) – from plants grown in plastic-covered experimental greenhouses (Zeraim Iberica S.A./Syngenta Seeds, Ltd., El Ejido, Almería, Spain). Supplementary Figure S1 shows a typical distribution of fruits at the three stages mentioned above (G, BP, and R) in pepper plants. In addition, to study the exogenous NO gas effect on the fruit ripening, we set two additional groups: treated fruits with 5 ppm NO for 1 h (BP2 + NO) and another parallel group that was not treated with NO (BP2 − NO) (Palma et al., 2018; González-Gordo et al., 2019). After 3 days, all fruits from the five groups were chopped into small cubes (5 mm/edge), frozen under liquid nitrogen, and then stored at −80°C until use. Figure 1 provides a comprehensive picture of the experimental design used in this study, which is based on a previous report (González-Gordo et al., 2019).

### Preparation of Pepper Samples for Biochemical Analyses

The pericarp of pepper fruits stored at −80°C of the different groups were ground under liquid nitrogen in an IKA A11 Basic mill and then dissolved in a ratio 1:1 (w/v) with 50 mM of Tris-HCl buffer, pH 7.5, 0.1 mM of EDTA, 0.1% (v/v) Triton X-100, 10% (v/v) glycerol, and 5 mM of dithiothreitol (DTT). In all cases, pericarp samples were prepared from, at least, five fruits (biological replicates) at each stage and treatments. Homogenates were filtered through two layers of Miracloth and centrifuged at 27,000 × *g* at 4°C for 30 min. The supernatants were used for the enzymatic assays.

### In-Gel Isozyme Profile of NADPH Oxidase and Superoxide Dismutase Activity Assays

Protein samples were separated using non-denaturing polyacrylamide gel electrophoresis (PAGE) on 6% acrylamide gels, and O_2_^•–^-generating NOX isozymes were visualized by a photochemical nitro blue tetrazolium (NBT) reduction method described previously (Airaki et al., 2012; Chu-Puga et al., 2019). Briefly, after the electrophoresis, the gels were incubated in the dark for 20 min in a reaction mixture solution containing 50 mM of Tris-HCl buffer (pH 7.4), 0.2 mM of NBT, 0.1 mM of MgCl_2_, and 1 mM of CaCl_2_. Subsequently, 0.2 mM of NADPH was added, and the appearance of the blue formazan bands was monitored. The reaction was stopped by immersing the gels in distilled water. As controls, gels were preincubated 30 min with 50 μM of diphenyleneiodonium (DPI), a specific inhibitor of NADPH-dependent O_2_^•–^ generation activity (Leterrier et al., 2012).

Superoxide dismutase (SOD) isozymes were separated by non-denaturing PAGE on 10% acrylamide gels, and activity was localized in gels by the NBT reduction method with O_2_^•–^ radicals generated photochemically (Beauchamp and Fridovich, 1971). To identify the type of SOD isozymes, the gels were preincubated separately at 25°C for 30–45 min in 50 mM of K-phosphate, pH 7.8, in the presence or absence of either 5 mM of KCN or 5 mM of H_2_O_2_. CuZn-SOD is inhibited by KCN and H_2_O_2_; Fe-SOD is inhibited by H_2_O_2_ but not by KCN, and Mn-SOD is not inhibited by either KCN or H_2_O_2_ (Corpas et al., 1998).

For the *in vitro* assay of the effect of some RNS and oxidizing/reducing agents on the isozyme SOD activity, pepper samples were preincubated with different chemicals including 4 mM of 3-morpholinosydnonimine (SIN-1), a peroxynitrite (ONOO^–^) donor, 4 mM of *S*-nitrosoglutathione (GSNO) and 4 mM of diethylamine NONOate (DEA NONOate) as NO donors, 10 mM of H_2_O_2_ as an oxidant; 4 mM of reduced glutathione (GSH), and 10 mM of DTT as reductants. All preincubations were done at 25°C for 2 h in the dark. Then, electrophoresis gels were stained for SOD activity as it has been previously described. In certain assays as those described in Figure 7, gels were preincubated for 30 min under dark conditions with either 5 mM of H_2_O_2_ or 5 mM of GSH before staining for SOD activity.

Protein concentration was determined using the Bio-Rad protein assay (Hercules, CA, United States), with bovine serum albumin as standard. Band intensity of RBOH isozymes was quantified using ImageJ 1.45 software^1^.

### RNA Extraction, Sequencing, and Real-Time Quantitative PCR

Total RNA was isolated from pepper fruits using a two-step method based on TRIzol^®^ Reagent (Gibco BRL) and the RNAeasy Plant Mini Kit (Qiagen), following the manufacturer’s instructions. Libraries were prepared using an optimized Illumina protocol and were sequenced on an Illumina NextSeq550 comprising four independent replicates belonging to the green stage and five to each of the other stages (González-Gordo et al., 2019). RNA (1 μg) was used for cDNA synthesis. It was added to a mixture containing 0.67 mM of dNTPs and 5 μM of oligo d(T)23VN in a volume of 15 μl. This mix was incubated 5 min at 65°C. Afterward, reaction buffer [75 mM of Tris-HCl, pH 9.0, 2 mM of MgCl_2_, 50 mM of KCl, and 20 mM of (NH_4_)_2_SO_4_], 1 U/μl of RnaseOUT^TM^ (Invitrogen), and 10 U/μl of SuperScript^TM^ II Reverse Transcriptase (Invitrogen) were added in a final reaction volume of 20 μl. Finally, cDNA synthesis was performed at 42°C for 50 min, followed by an inactivation step of 15 min at 70°C.

In order to get our reference transcriptome and differentially expressed (DE) genes among the ripening stages and the NO treatment, we analyzed 24 biological replicates (4 × G, 5 × BP1, 5 × BP2 + NO, 5 × BP2 − NO, and 5 × R) and followed an optimized workflow to process all the data. Data processing involved several bioinformatic tools (TransFlow, Seoane et al., 2018 and DEgenes Hunter, González-Gayte et al., 2017), which apply different algorithms with their own statistical tests, to validate the whole experiment. The trends found in the expression patterns in the RNA-Seq experiments were confirmed by performing the real-time quantitative PCR (RT-qPCR) experiments.

Real-time quantitative PCR experiments were performed on a QuantStudio 3 Real-Time PCR System (Applied Biosystems, Foster City, CA, United States) with QUANTIMIX HotSplit Easy kit (Biotools, B&M Labs, Madrid, Spain) following manufacturer’s instructions and using specific primers (see Supplementary Table S1). *Actin* and *glyceraldehyde−3−phosphate dehydrogenase* (*GAPDH*) were used as housekeeping genes (Wan et al., 2011; Bin et al., 2012). Reactions were performed with an initial step at 95°C for 30 s and then cycled at 95°C for 1 min, 67°C for 1 min, and 72°C for 15 s for 40 cycles. Each PCR was performed at least three times (technical replicates), with three independent samples (biological replicates). Relative gene expression was calculated using the 2^–ΔΔCt^ method (Schmittgen and Livak, 2008). A time-course expression analysis of *RBOH* and *SOD* genes constitutes a trustworthy visual representation of the expression values obtained by RNA-Seq.

### Phylogenetic Analysis and Conserved Respiratory Burst Oxidase Homolog Protein Sequences

Alignment of plant RBOH proteins was performed using the ClustalW tool with default parameters. Then, a phylogenetic tree was generated using MEGA 7.0 and edited with Figtree software. Sequence logos of conserved motifs were created by WebLogo 3.

### Statistical Analysis

A one-way analysis of variance (ANOVA) was used to test the statistical significance between relative expression values obtained using RT-qPCR. *Post hoc* comparisons of means were made by using a Tukey honestly significant difference (HSD) test. Statistical significance was considered at the conventional 5% level (*P* ≤ 0.05). All calculations were performed using R Studio.

## Results

The main goal of this study is to understand how O_2_^•–^ metabolism is modulated during pepper fruit ripening with special focus in the activity of two groups of isozymes, O_2_^•–^-generating NOXs and the antioxidant SODs. Additionally, it was evaluated how these two enzymatic systems could be affected in the presence of a NO-enriched environment during the ripening of sweet pepper fruits.

**FIGURE 2:**
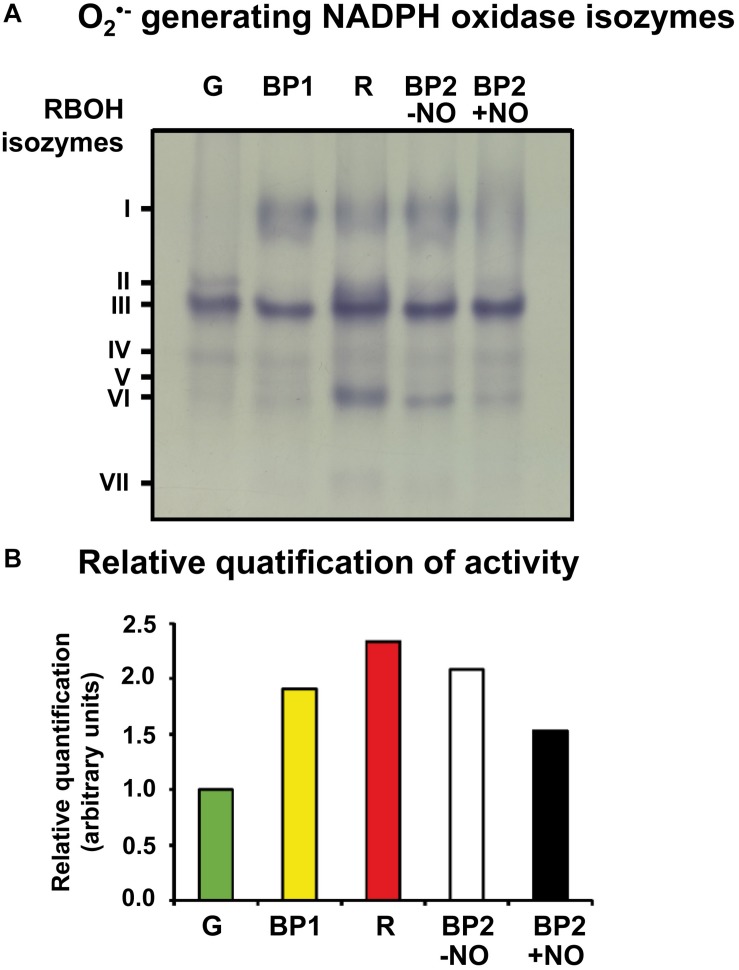
Activity **(A)** and relative quantification **(B)** of O_2_^–^ -generating NADPH oxidase isozymes of sweet pepper fruits at different stages of ripening: immature green (G), breaking point 1 (BP1), breaking point 2 with and without NO treatment (BP2 + NO and BP2 – NO, respectively), and red ripe (R). Protein samples (25 μg per lane) were separated by non-denaturing polyacrylamide gel electrophoresis (PAGE; 6% acrylamide), and activity was detected by the nitro blue tetrazolium (NBT)-reducing method. Isozymes were labeled I–VII (on the left) according to their increasing electrophoretic mobility. Comparative total band intensities in each lane were quantified using ImageJ 1.45 software (see Supplementary Figure S1 for details). The gel image is representative of those obtained from at least three biological replicas repeated three times.

With the use of these group of samples, Figure 2A shows the O_2_^•–^-generating NOX isozymes profile of sweet pepper fruits at different stages of ripening: immature green, BP1, BP2 with and without NO (BP2 + NO and BP2 − NO, respectively), and red. Thus, a total of seven O_2_^•–^-generating NOX isozymes, with different electrophoretic mobility and abundance, were globally detected during the ripening from green to red fruits considering all the different stages and the NO treatment. They were designated as I to VII according to their increasing electrophoretic mobility. Supplementary Figure S2 illustrates the relative quantification of each O_2_^•–^-generating NOX isozyme in the analyzed ripening stages, with isozyme III being the most prominent in all ripening stages. Figure 2B shows the total relative quantification considering all the O_2_^•–^-generating NOX isozymes present in each ripening stages. Thus, a gradual increased activity was observed throughout ripening with a maximum of about 2.4-fold increase in red fruit compared with green fruit. Significantly, pepper fruits both with and without NO treatment (BP2 + NO and BP2 − NO) showed intermediate values to those observed for green and red peppers (Figure 2B), with the value in NO-treated fruits being lower than in untreated fruits. In this case, isozymes I and VI seemed to be the most affected.

**FIGURE 3:**
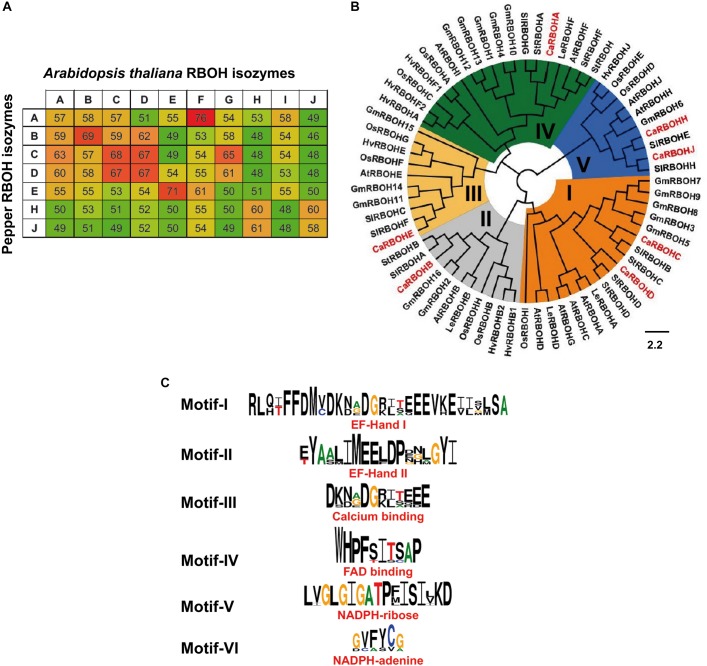
Analyses of sweet pepper respiratory burst oxidase homolog (RBOH) isozymes based on their protein sequences. **(A)** Percentage of protein sequence identity among the 10 *Arabidopsis* RBOH isozymes with the seven sweet pepper RBOH isozymes. High, intermediate, and low identities are shown as green, yellow, and red color, respectively. **(B)** Phylogenetic analysis and conserved regions of sweet pepper RBOH proteins. Maximum-likelihood tree of 67 plant RBOH protein sequences. Identified CaRBOH proteins are highlighted in red color. The scale bar represents the phylogenetic branch length. Different subgroups of RBOHs are depicted in different colors (I–V). Species abbreviations: At (*Arabidopsis thaliana*), Ca (*Capsicum annuum*), Gm (*Glycine max*), Hv (*Hordeum vulgare*), Ls (*Lepidium sativum*), Os (*Oryza sativa*), Sl (*Solanum lycopersicum*), and St (*Solanum tuberosum*). **(C)** Conservation of sequence motifs (I–VI) of sweet pepper RBOH protein sequences.

To get deeper knowledge about this O_2_^•–^-generating NOX isozymes during ripening, the identification of *RBOH* genes in the transcriptome of sweet pepper previously reported (González-Gordo et al., 2019) and their corresponding protein sequences were accomplished. With this information, one could make a comparative analysis of the percentage of identities among the protein sequences of the 10 *Arabidopsis* RBOH isozymes (A to J) with the seven identified sweet pepper RBOH isozymes because *Arabidopsis thaliana* provides the most complete information on this subject (Figure 3A). Considering the highest degree of protein identities with respect to the *Arabidopsis* RBOHs, the seven sweet pepper RBOH isozymes identified here were designated with letters A, B, C, D, E, H, and J.

Figure 3B illustrates the phylogenetic comparative analysis among RBOH proteins from eight plant species including sweet pepper (*Capsicum annuum*), *Arabidopsis thaliana*, garden cress (*Lepidium sativum*), soybean (*Glycine max*), barley (*Hordeum vulgare*), rice (*Oryza sativa*), and two species of the *Solanum* genus: tomato (*Solanum lycopersicum*) and potato (*Solanum tuberosum*). This allowed to identify five main RBOH groups designated as I to V, which are depicted with different colors in the diagram. CaRBOH A, reported in this work, was included in group IV, CaRBOH B was present in group II, CaRBOH C and D are in group I, CaRBOH E is in group III, and CaRBOH H and J are in group V. Figure 3C shows the identification of six conserved motifs of plant RBOHs present in sweet pepper RBOH protein sequences, which correspond to EF-Hand I and II, calcium and FAD binding, NADPH-ribose, and NADPH-adenine motifs, respectively.

**FIGURE 4:**
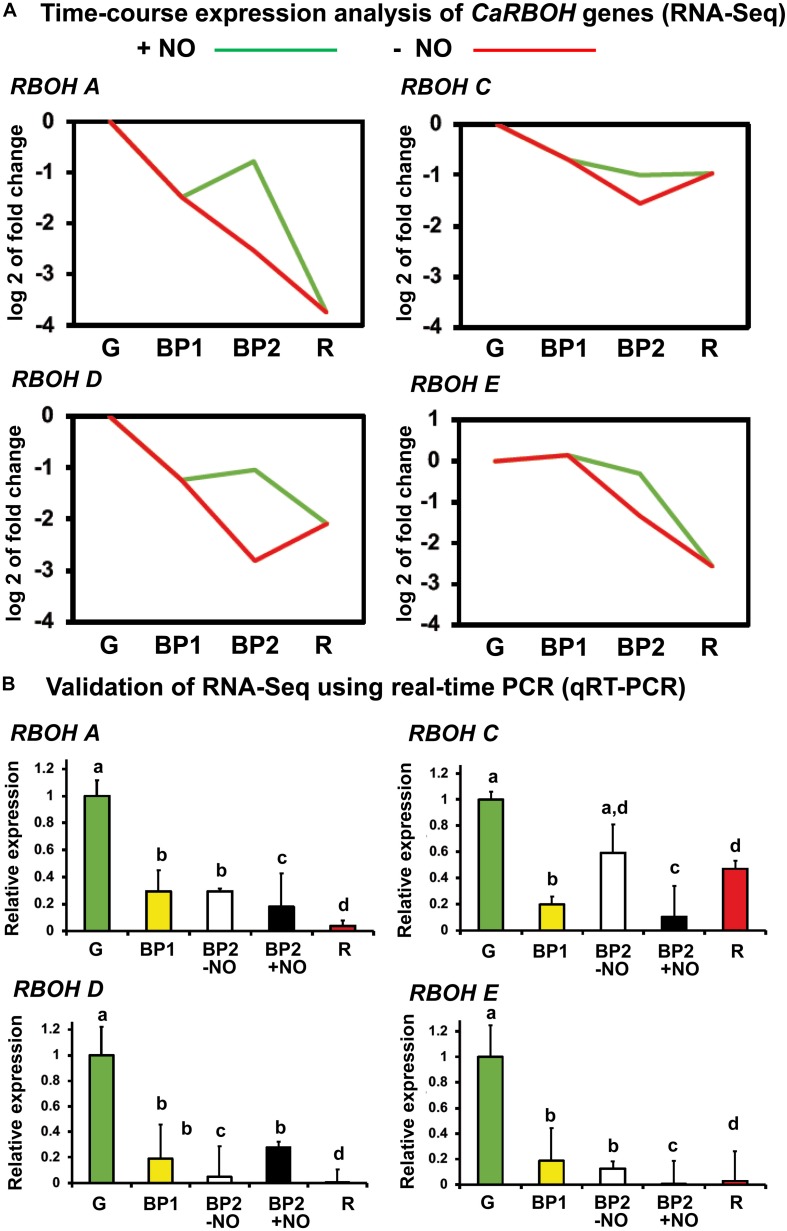
**(A)** Time-course expression analysis of four *CaRBOH* genes (RNA-Seq). **(B)** Validation of RNA-Seq data using real-time quantitative PCR (RT-qPCR). Samples of sweet pepper fruits at different stages of ripening correspond to immature green (G), breaking point 1 (BP1), and breaking point 2 with and without NO treatment (BP2 + NO and BP2 – NO, respectively). Each PCR was performed at least three times, with three independent samples. Different letters indicate significant differences (*P* < 0.05).

With the seven identified *RBOH* genes in the sweet pepper transcriptome (RNA-Seq) (González-Gordo et al., 2019), a time-course analysis was done, and among these seven genes, only the designated *RBOH A*, *C*, *D*, and *E* were found to show expression changes in our experimental design. Figure 4A shows the time-course expression analysis of these four *RBOH* genes by RNA-Seq of sweet pepper fruits at different stages of ripening mentioned before including the NO treatment. In all four cases, the *RBOH* gene expression was downregulated from green (G) to red (R) stage. In general, the gene expression in fruits with and without NO treatment (BP2 + NO and BP2 − NO, respectively) both showed intermediate values between those observed for green and red peppers, but in all cases, the NO treatment increased the expression of the four *RBOH* genes. To corroborate these data, RT-qPCR analyses of the genes were done using the same fruit sample set to perform the RNA-Seq approach (Figure 4B). Thus, the expression of the four *RBOH* genes was also downregulated throughout ripening. Additionally, the NO effect (BP2 + NO) in the gene expression did not show significant difference regarding the BP − NO group, with the exception of *RBOH C* expression, which was downregulated.

**FIGURE 5:**
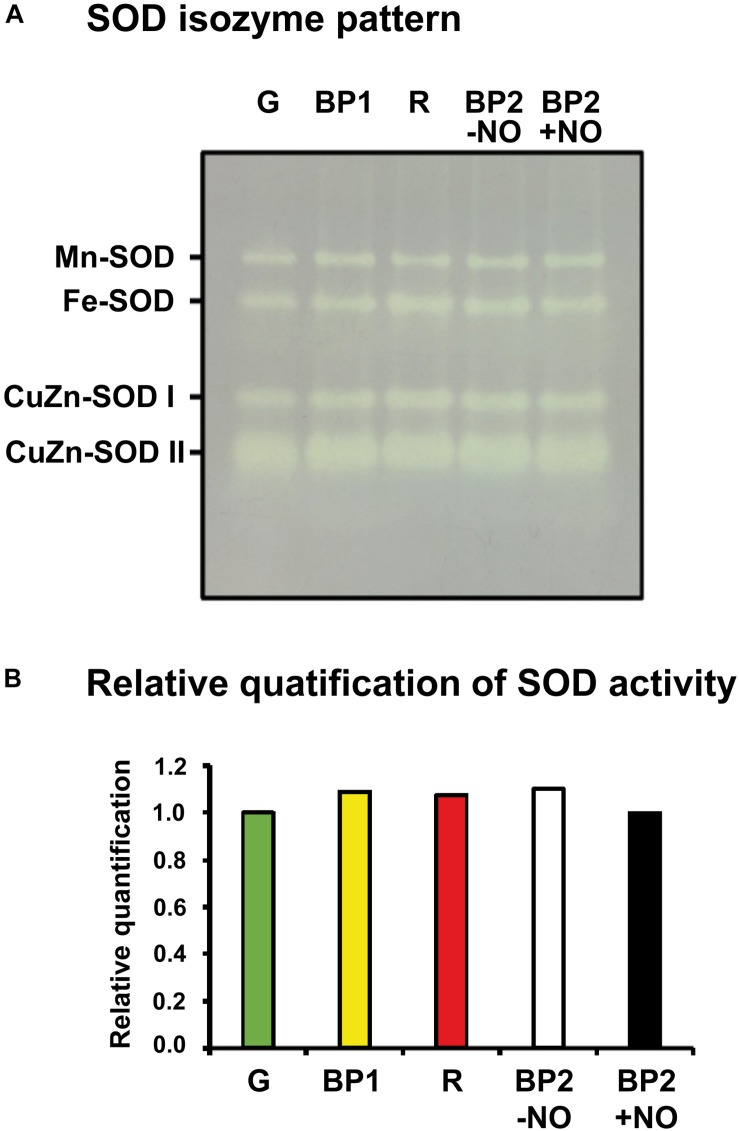
Activity **(A)** and relative quantification **(B)** of superoxide dismutase (SOD) isozymes of sweet pepper fruits at different stages of ripening: immature green (G), breaking point 1 (BP1), and breaking point 2 with and without NO treatment (BP2 + NO and BP2 – NO, respectively). Protein (5 μg per lane) samples were separated by non-denaturing polyacrylamide gel electrophoresis (PAGE; 8%), and the activity was detected by the nitro blue tetrazolium (NBT)-reducing method. Comparative total band intensities were quantified using ImageJ 1.45 software (see Supplementary Figure S1 for details). The gel pictures are representative of those obtained from at least three biological replicas repeated three times.

As part of the O_2_^•–^ metabolism analysis, the SOD system activity and its gene expression during pepper fruit ripening were also studied. Figure 5A depicts the SOD isozyme profile in non-denaturing polyacrylamide gel of sweet pepper fruits at the different ripening stages used in this work: immature green (G), BP1, BP2 with and without NO treatment (BP2 + NO and BP2 − NO, respectively), and ripe red (R). According to the mobility in gels and the response to inhibitors, a total of four SOD isozymes were identified: one Mn-SOD, one Fe-SOD, and two CuZn-SODs. It is remarkable to point out that after the quantification of the SOD in-gel activity, none of the isozymes showed any significant change either during the ripening from green to red fruits or considering the different stages after the NO treatment (Figure 5B).

**FIGURE 6:**
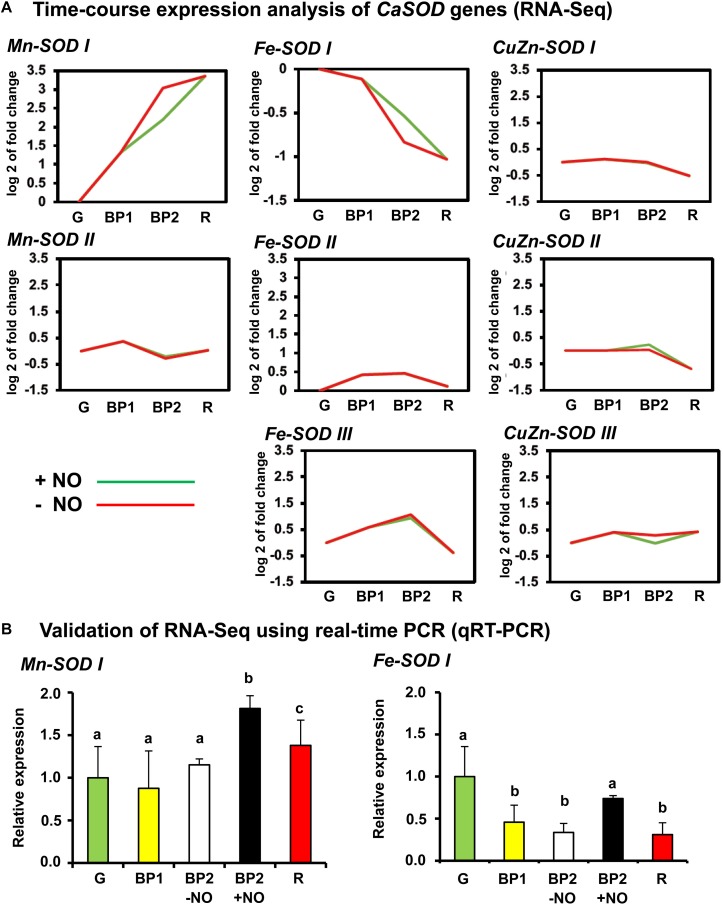
**(A)** Time-course expression analysis of *CaSOD* genes (RNA-Seq). **(B)** Validation of RNA-Seq for *Mn-SOD I* and *Fe-SOD I* genes using real-time quantitative PCR (RT-qPCR). Each PCR was performed at least three times, with three independent samples. Different letters indicate significant differences (*P* < 0.05).

The search of *SOD* genes in the sweet pepper transcript database previously obtained by RNA-Seq (González-Gordo et al., 2019) allowed finding a total of eight *SOD* genes including two *Mn-SOD*s, three *Fe-SOD*s, and three *CuZn-SOD*s. Figure 6A shows the time-course expression analysis of these eight *SOD* genes (RNA-Seq) of sweet pepper fruits at the different ripening stages reported above. Among these eight *SOD* genes, only two, *Mn-SOD I* and *Fe-SOD I*, were modulated during ripening and under the NO treatments. Whereas the *Mn-SOD I* expression increased during ripening, the *Fe-SOD I* showed an opposite behavior. Additionally, under NO treatment, the expression of these two genes was also different because *Mn-SOD I* was downregulated after NO treatment whereas *Fe-SOD I* expression increased. The data of the two genes obtained by RNA-Seq were corroborated by RT-qPCR (Figure 6B).

**FIGURE 7:**
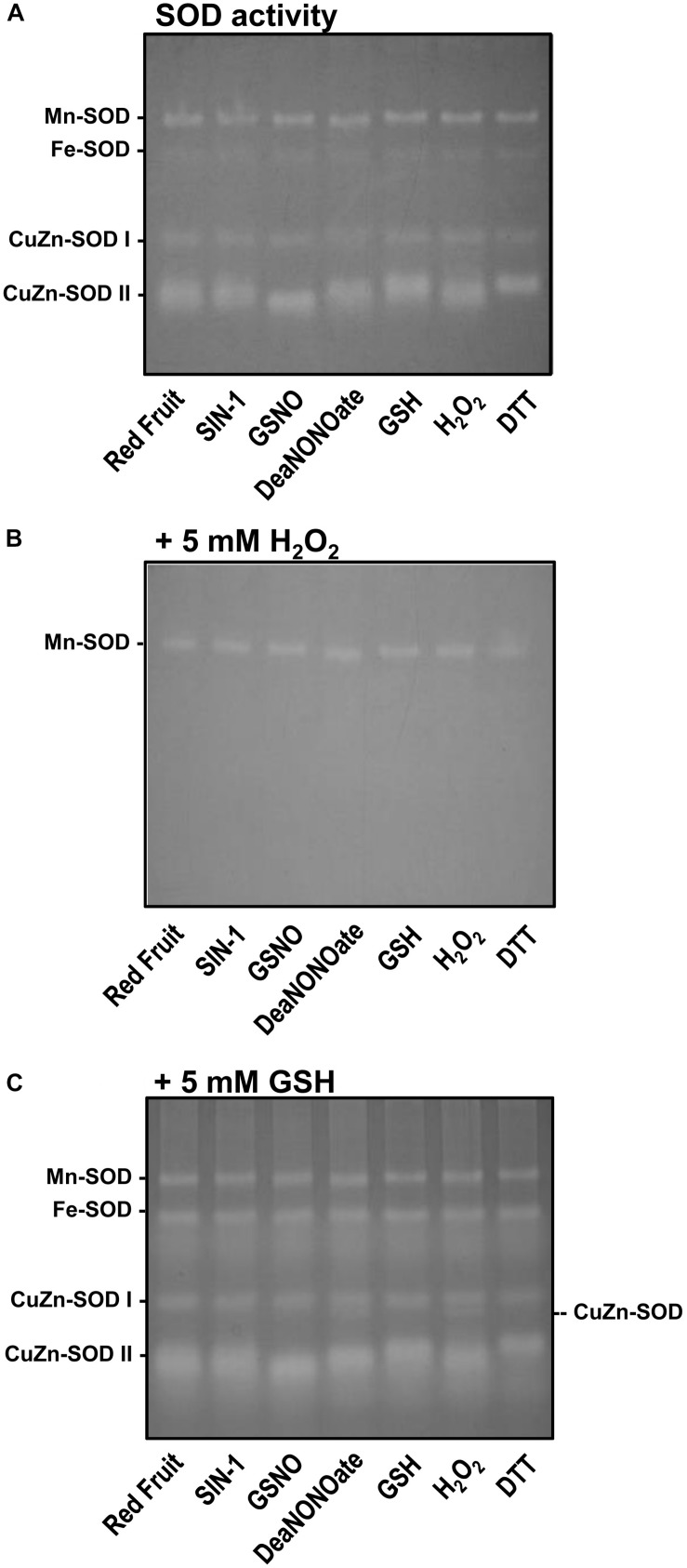
Analysis on gel electrophoresis of the activity of the different superoxide dismutase (SOD) isozymes of red pepper fruit samples preincubated with different chemicals. **(A)** Samples were preincubated with 4 mM of SIN-1, 4 mM of GSNO, 4 mM of DEA NONOate, and 4 mM of GSH; 10 mM of H_2_O_2_; and 10 mM of DTT. After electrophoresis (acrylamide 8%), gels were stained for SOD activity. **(B)** Samples were processed as in panel **(A)**, but the gel was preincubated with 5 mM of H_2_O_2_ before staining for SOD activity. **(C)** Samples were processed as in panel **(A)**, but the gel was preincubated with 5 mM of GSH prior to SOD activity staining. The gel pictures are representative of those obtained from at least three biological replicas repeated three times.

In a previous study, we showed that in sweet pepper, the O_2_^•–^ generation by a NOX system was inhibited in the presence of NO donors, ONOO^–^, and GSH, suggesting that the responsible enzymes could be regulated by *S*-nitrosation, Tyr-nitration, or glutathionylation, respectively (Chu-Puga et al., 2019). However, to our knowledge, there is no such similar analysis of SOD isozymes from pepper fruits. Consequently, we performed a series of biochemical *in vitro* assays of the SOD activity in the presence of different chemicals including 4 mM of SIN-1 as ONOO^–^ donor, 4 mM of GSNO and 4 mM of DEA NONOate as NO donor, 4 mM of GSH and 10 mM of DTT as reductants, and 10 mM of H_2_O_2_ as oxidant. Figure 7A shows that, except for the slight changes in the electrophoretic mobility of CuZn-SOD II, none of the SOD isozymes present in the red fruits were affected for any of the treatments. To discard potential masking effect, considering that both CuZn-SOD and Fe-SOD activities are well known to be inhibited by H_2_O_2_, it was decided to repeat the experiments, but in this case, the gel was incubated in the presence of 5 mM of H_2_O_2_ once the samples were incubated with the chemicals and the electrophoresis was completed. As observed in Figure 7B, and as expected, both CuZn-SODs and Fe-SOD were indeed inhibited whereas the Mn-SOD was unaffected in all cases. With the goal to get deeper knowledge about SOD isozyme activities and their potential regulation by RNS and distinct redox conditions, a similar analysis was done, but in this case, the gel was incubated with 5 mM of GSH (Figure 7C). In this situation, it was observed that none of the SOD isozymes underwent significant changes in its activity. However, we detected a new CuZn-SOD band close to and faster than the CuZn-SOD I in the samples treated with DEA NONOate and H_2_O_2_.

## Discussion

Reactive oxygen species generation is part of the cellular metabolism, and depending on the levels of production and the ROS type, they could have either toxic effects or signal properties. There are evidences that ROS metabolism is involved in a diverse range of plant processes, and the available data indicate that fruit development and ripening have an active ROS metabolism in different species such as grapevine berry (Pilati et al., 2014), mango (Rosalie et al., 2015), peach (Huan et al., 2016), tomato (Kumar et al., 2016; Zhu et al., 2017), grape berry (Xi et al., 2017), or guava (Mondal et al., 2009; Batista-Silva et al., 2018). In previous reports on sweet pepper ripening, we focused on the H_2_O_2_ metabolism, and it was found that catalase activity and their gene expression were diminished during ripening. Moreover, catalase was downregulated by NO-mediated posttranslational modifications including Tyr-nitration and *S*-nitrosation (Chaki et al., 2015; Rodríguez-Ruiz et al., 2019). To get deeper knowledge on the ROS metabolism during sweet pepper ripening, the present study has been focused on another main ROS, specifically in O_2_^•–^, to analyze some of the biochemical players involved in their generation and decomposition. With the goal to get a deeper understanding of the ROS metabolism during sweet pepper fruit ripening, especially on the metabolism of O_2_^•–^ and its potential modulation under a NO-enrichment environment, we have based on a previous study where we reported the first transcriptome of sweet pepper at different ripening stages, being also compared with the transcriptome under a NO-enrichment environment (González-Gordo et al., 2019). In such study, it was observed that during fruit ripening the ROS metabolism was altered with an increase of lipid oxidation and that NO reduced this parameter with a concomitant increase in the content of GSH and ascorbate peroxidase (APX) activity.

### O_2_^•–^ Generation by a NADPH-Dependent Oxidase System Increases During Ripening but Is Negatively Modulated by NO

It should be mentioned that to our knowledge there are only few reports focused on the function of a specific RBOH activity during fruit ripening. In an early study, we found up to seven NADPH-oxidase isozymes that were differently regulated during the ripening of sweet pepper fruits (Chu-Puga et al., 2019). It should be remarked that the identification of the seven isozymes in non-denaturing gel activity assays involved the optimization of the amount of loaded protein samples for each stage being determined in green fruit by almost two-fold the amount found in red fruits. Thus, these seven NADPH-oxidase isozymes with different electrophoretic mobility and abundance were detected considering both green and red fruits. The present data corroborate those previous data, but the analysis of the intermediate ripening stages in the presence of NO (BP2 + NO) showed a fine regulation of this system because isozymes I and III displayed lower activities when they were compared with the BP2 without NO treatment (BP2 − NO); however, one cannot discard the existence of additional isozymes whose activity may be too low for their detection in our experimental conditions. To our knowledge, the only identified NOX that has been reported to be negatively regulated by NO corresponds to the *Arabidopsis* RBOH isozyme D activity (AtRBOHD) that was inhibited by *S*-nitrosation at Cys825, thus disturbing the mechanism of the immune response (Yun et al., 2011). Very recently, it has been reported that this AtRBOHD is also susceptible to undergo persulfidation, a posttranslational modification mediated by hydrogen sulfide (H_2_S) (Corpas et al., 2019), at Cys825 and Cys890, provoking an increase in the O_2_^•–^ production, which was relevant in the ABA signaling to induce stomatal closure (Shen et al., 2020). In pepper fruit, we have previously described that the content of endogenous H_2_S increases at ripening (Muñoz-Vargas et al., 2018) and that it could be hypothesized that this H_2_S might be involved in the observed increase of NOX activity. However, future analyses should be carried out to corroborate this and to identify what isozyme(s) could be the target of persulfidation in our plant model.

On the other hand, these data indicate that this increase in NOX activity might be necessary in the physiological ripening process, and this is concomitant with the enhanced NADPH-generating systems described during sweet pepper ripening, specifically NADP-malic enzyme and 6-phosphogluconate dehydrogenase activities (Muñoz-Vargas et al., 2020). However, the O_2_^•–^ generation could have other beneficial effects in pepper fruit as a mechanism to prevent potential pathogen infections (Tian et al., 2013). In this sense, the latest results in apricot fruit treated exogenously with polyamines showed that the activities and gene expression levels of NOX systems were triggered, allowing the resistance to the fungus *Alternaria alternata* (Li et al., 2019). Recently, it has been described by *in silico* analyses the identification of the *RBOH* family genes in Chinese white pear (*Pyrus bretschneideri*), which contains 10 *RBOH*s. Among these genes, it was found that *PbRBOHA* and *PbRBOHD* were specially accumulated in pear stone cells, suggesting the involvement of ROS metabolism in the lignification of these cells (Cheng et al., 2019).

### Sweet Pepper Fruit Transcriptome Contains Seven Respiratory Burst Oxidase Homolog Isozymes

Based on the comparative analysis between the *Arabidopsis RBOH* genes with the sweet pepper fruit transcriptome (González-Gordo et al., 2019) and their corresponding protein sequences, the seven pepper RBOH proteins were designated with the letters A, B, C, D, E, H, and J. All these RBOH proteins showed the six characteristic conserved motifs of plant RBOHs, which correspond to EF-Hand I and II, calcium and FAD binding sites, NADPH-ribose, and NADPH-adenine (Torres and Dangl, 2005; Kaur et al., 2018). However, the correspondence between the seven NOX isozymes identified with the seven *RBOH* genes reported here need to be confirmed by future experiments where each pepper RBOH protein encoded by its respective gene needs to be purified, and their mobility needs be evaluated under non-denaturing gel electrophoresis.

### There Is No Correlation Between NADPH Oxidase and Superoxide Dismutase Isoenzymatic Activities and the Corresponding Genes

In our experimental model of sweet pepper, it should be pointed out that the analyses of activity and gene expression of both NOX and SOD isozymes have different behaviors. Thus, it was found that whereas O_2_^•–^ generation by NOX isoenzymes increased during ripening, the identified *RBOH* genes were downregulated. Comparative observation could be made with the activity of SOD isozymes, which did not show any significant variations, whereas the gene expression of *Mn-SOD* increased, *Fe-SOD* decreased, and *CuZn-SOD* did not change. This apparent contradiction between enzyme activity and gene expression is not unusual, and there are numerous examples where the inexistent correlation between gene expression and their corresponding protein activity has been described. Recently, the analysis of protein and transcript abundance during tomato fruit development and ripening showed a poor correlation owing to, among other factors, the rate of protein translation and degradation; in fact, the levels of transcripts were more reduced than the protein levels (Belouah et al., 2019). Among other factors that could support these discrepancies, the protein posttranslational modifications including those mediated by ROS or RNS could be considered.

### The Superoxide Dismutase Isoenzymatic Pattern Was Unaffected Either During Ripening or by a NO-Enriched Environment

In a previous study, we showed by *in vitro* analyses that the O_2_^•–^ generation by the NADPH-oxidase system was inhibited in the presence of NO donors, ONOO^–^, and GSH, suggesting that the responsible enzymes can undergo *S*-nitrosation, Tyr-nitration, and glutathionylation, respectively (Chu-Puga et al., 2019). Although we did not observe any apparent change in the SOD isoenzymatic activity during ripening or in the NO-enriched atmosphere, we decided to prepare *in vitro* analyses to study the pepper SOD isoenzymatic activity because in the *in vitro* analysis of the seven *Arabidopsis* SOD isozymes, it was reported that, using GSNO as NO donor, none of the *Arabidopsis* SODs were affected. Conversely, peroxynitrite (a nitrating agent) triggered the inhibition of the mitochondrial Mn-SOD, peroxisomal CuZn-SOD (CSD3), and chloroplastic iron SOD3 (FSD3), whereas the other SODs were unaffected (Holzmeister et al., 2015). In our experimental conditions of sweet pepper, neither GSNO nor peroxynitrite seems to affect any of the SOD isozymes. Nevertheless, it was found that the pretreatment of pepper sample with NO and H_2_O_2_ previous to the gel analysis of the SOD isozymes allowed to identify a new CuZn-SOD with mobility higher than that of the CuZn-SOD I but slower than that of CuZn-SOD II. A reasonable explanation of the appearance of a new isoenzyme could be due to the presence of two different oxidation states of the CuZn-SOD I corresponding to a mixed Cu(I)/Cu(II) redox pair after these mentioned treatments, such as it has been described in bovine and human CuZn-SOD after isoelectrofocusing analyses (Chevreux et al., 2009). A similar behavior of the potential oxidation of a CuZn-SOD that affected the electrophoretic mobility of the enzyme was also reported for the cytosolic CuZn-SOD from watermelon cotyledons (Palma et al., 1997).

## Conclusion

Reactive oxygen species metabolism is of great importance for fruit ripening because it is involved in the process of development and quality (Decros et al., 2019). The present study has focused on the O_2_^•–^ metabolism in the non-climacteric sweet pepper ripening, and the data support that O_2_^•–^-generating NOX production increases during ripening being regulated by NO. On the other hand, the SOD activity of the different isoenzymes was unaffected during ripening, and it should be pointed out that none of the SOD isozymes were significantly affected by any of the assayed *in vitro* nitro-oxidative conditions. This suggests that the basal SOD activity is sufficient to keep the homeostasis of the necessary physiological O_2_^•–^ production during sweet pepper ripening. This prevalence of O_2_^•–^ production could have additional benefits because they could be a barrier to prevent potential pathogen infections. Moreover, these new data of the prevalence of O_2_^•–^-generating NOX during sweet pepper fruit ripening should be also integrated with the NADPH-generating system constituted by a group of NADP-dehydrogenases, which are regulated by signal molecules such as NO and H_2_S (Muñoz-Vargas et al., 2018, 2020). Additionally, future analyses should be developed to understand the integration of the redox homeostasis with the phytohormones including ABA, indole-3-acetic acid, gibberellins, cytokinins, or jasmonic acid involved in the ripening of sweet pepper considering that it is a non-climacteric fruit (Symons et al., 2012; Fuentes et al., 2019; Sun et al., 2019). This will allow to extend our knowledge of the complex network of involved signal molecules in this physiological process.

## Data Availability Statement

The datasets generated for this study are available on request to the corresponding author.

## Author Contributions

FC and JP conceived and designed the experiments. SG-G and MR-R performed the experiments. SG-G, JP, and FC analyzed the data. All authors contributed to drafting the work, revised the final manuscript, and approved submission.

## Conflict of Interest

The authors declare that the research was conducted in the absence of any commercial or financial relationships that could be construed as a potential conflict of interest.

## Footnotes

1 https://imagej.nih.gov/ij/
