# Supplementary figures and images for: Superoxide Radical Metabolism in Sweet Pepper (Capsicum annuum L.) Fruits Is Regulated by Ripening and by a NO-Enriched Environment
Source: Front Plant Sci. 2020 May 14;11:485. doi: 10.3389/fpls.2020.00485 (PMC7240112; doi:10.3389/fpls.2020.00485)

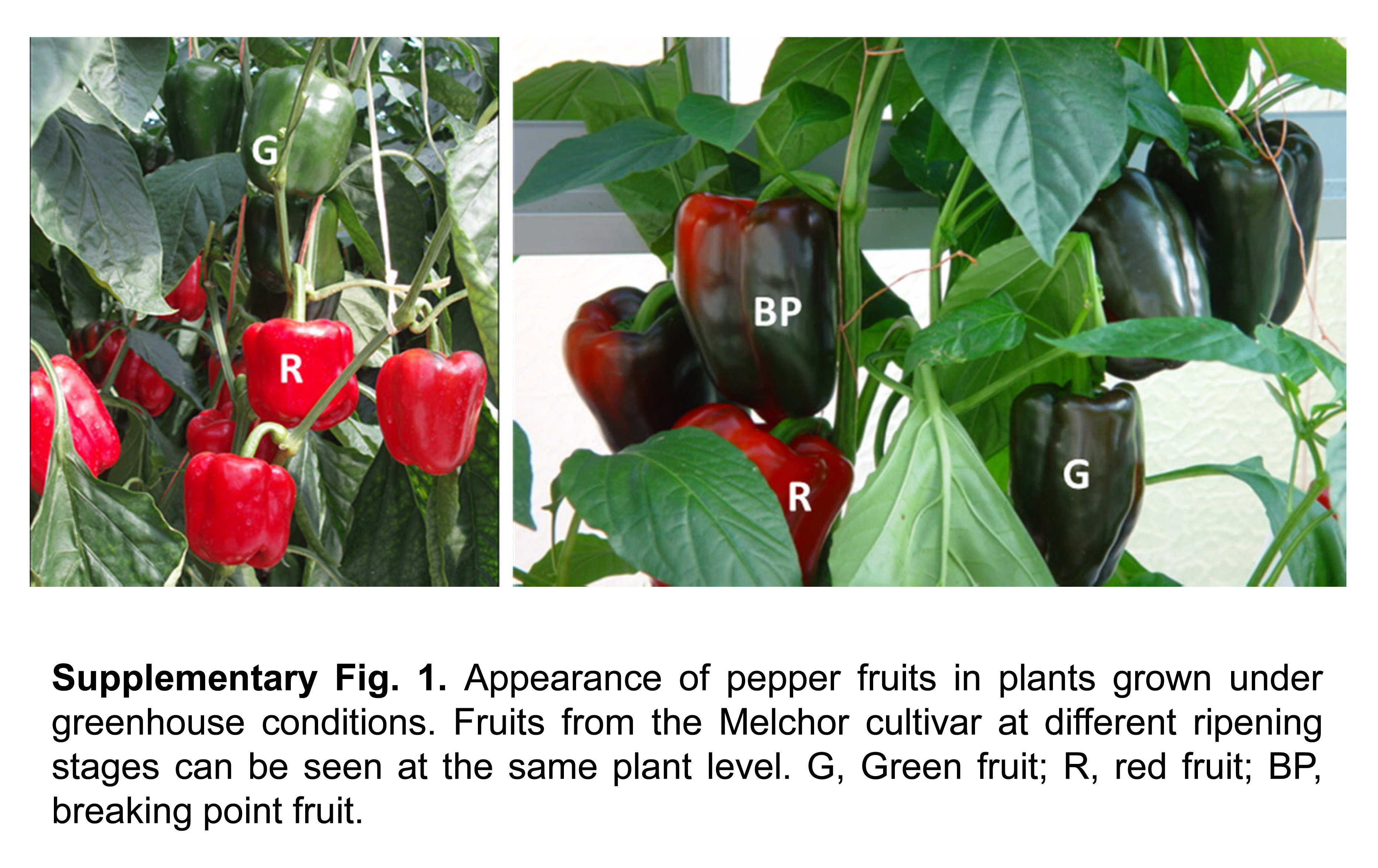

Supplement: Supplementary file 1 [file Image_1.JPEG]

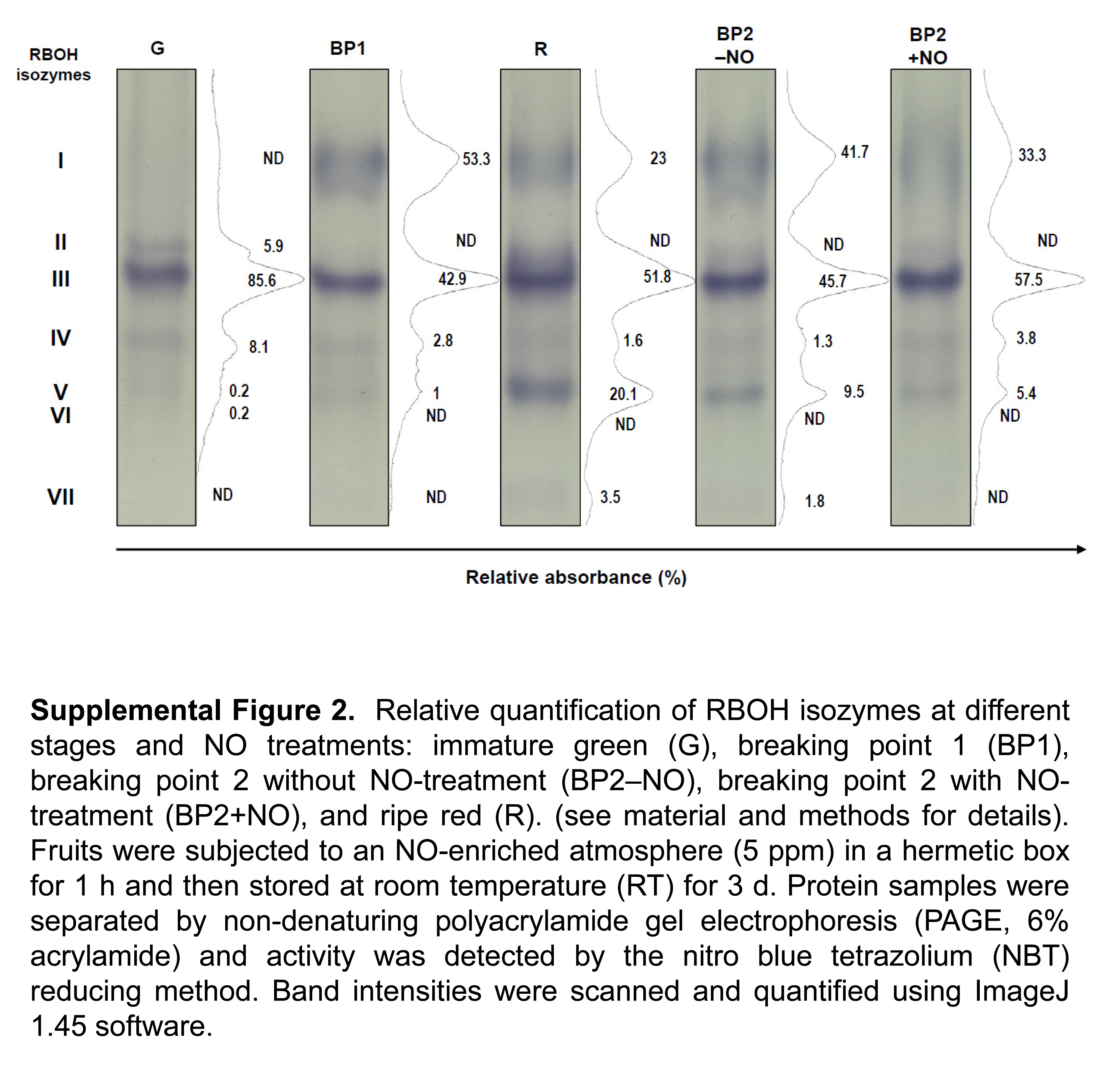

Supplement: Supplementary file 2 [file Image_2.JPEG]
