# Supplementary material for: Superoxide Radical Metabolism in Sweet Pepper (Capsicum annuum L.) Fruits Is Regulated by Ripening and by a NO-Enriched Environment
Source: Front Plant Sci. 2020 May 14;11:485. doi: 10.3389/fpls.2020.00485 (PMC7240112; doi:10.3389/fpls.2020.00485)
Supplement: Supplementary file 3 [file Table_1.DOCX]

**Table S1.** Oligonucleotide primers used for qPCR expression studies of *RBOH* and *SOD* genes.

| **Gene name** | **Accession No** | **Oligonucleotide sequence (5´–3´)** | **Amplicon** |
| --- | --- | --- | --- |
| Q-*CaACTIN* | LOC107840006 | CAAACAGGTTTTAAAAGATGGCAGATGAAG  TCCTTTTGACCCATCCCTACCATAACAC | 172 |
| Q-*CaGAPDH* | LOC107845282 | CGACAACGAGTGGGGTTACA  CTTGCGCCAACTTCTGCATT | 113 |
| Q-*CaRBOHA* | LOC107864242 | TGCTTTCACTCTTGCAACGC  AAACGTGCCATGGATGACCA | 148 |
| Q-*CaRBOHC* | LOC107862088 | CGGAAAATCATCACCCGCAC  ACTGGCACTGTTTCCGATGT | 170 |
| Q-*CaRBOHD* | LOC107875997 | CGGCTCCTAAACGCTAGTCC  ACCGTTGGGTTGCTAGAGTG | 160 |
| Q-*CaRBOHE* | LOC107876452 | GAGGGTGATGCCAGGTCAAC  AGGCAACCCGCAGTAGAAGA | 189 |
| Q-*CaMn-SOD* | LOC107871142 | AACTCCGATGCGTCCAAACT  CATGAAGCTGTTGAAGGGCA | 156 |
| Q-*CaFe-SOD* | LOC107873096 | ATTTGGTTCTGGGTGGGCTT  ATAGTGAGGAGTGGCGCGTA | 162 |
